# Estimation of Genetic Diversity and Number of Unique Genotypes of Cassava Germplasm from Burkina Faso Using Microsatellite Markers

**DOI:** 10.3390/genes15010073

**Published:** 2024-01-05

**Authors:** Monique Soro, Serge Marie Felicien Wend-Pagnagdé Zida, Koussao Somé, Fidèle Tiendrébéogo, Daniel H. Otron, Justin S. Pita, James B. Néya, Daouda Koné

**Affiliations:** 1 Central and West African Virus Epidemiology (WAVE), Pôle Scientifique et d’Innovation de Bingerville, Université Félix Houphouët-Boigny (UFHB), Bingerville 08 BP 2035, Côte d’Ivoire; danyotron452@gmail.com (D.H.O.); justin.pita@wave-center.org (J.S.P.); 2 Laboratoire de Virologie et de Biotechnologies Végétales, Institut de l’Environnement et de Recherches Agricoles (INERA), Ouagadougou 01 BP 476, Burkina Faso; koussao@hotmail.com (K.S.); neyajames@yahoo.fr (J.B.N.); 3 Laboratoire Mixte International Patho-Bios, Institut de l’Environnement et de Recherches Agricoles, Ouagadougou 01 BP 476, Burkina Faso; 4 Laboratoire de Génétique et de Biotechnologies Végétales, Institut de l’Environnement et de Recherches Agricoles (INERA), Ouagadougou 01 BP 476, Burkina Faso; felicienzida@yahoo.fr; 5 Laboratoire de Biotechnologie, Agriculture et Valorisation des Ressources Biologiques, UFR Biosciences, Université Félix Houphouët-Boigny, Abidjan 22 BP 582, Côte d’Ivoire; daoudakone2013@gmail.com; 6 Centre d’Excellence Africain sur le Changement Climatique, la Biodiversité et l’Agriculture Durable, Université Félix Houphouët-Boigny, Abidjan 22 BP 463, Côte d’Ivoire

**Keywords:** cassava, genotypes, genetic diversity, SSR markers, population structure, unique multilocus genotypes

## Abstract

Genetic diversity is very important in crop improvement. This study was carried out to assess the genetic diversity and the number of unique multilocus genotypes (MLGs) in a cassava collection in Burkina Faso. To achieve this objective, 130 cassava accessions were genotyped using 32 simple sequence repeat (SSR) markers. The results revealed that among these markers, twelve (12) were highly informative, with polymorphic information content (PIC) values greater than 0.50; twelve (12) were moderately informative, with PIC values ranging between 0.25 and 0.50; and eight (8) were not very informative, with PIC values lower than 0.25. A moderate level of genetic diversity was found for the population, indicated by the average expected heterozygosity (0.45) and the observed heterozygosity (0.48). About 83.8% of unique multilocus genotypes were found in the cassava collection, indicating that SSR markers seem to be most appropriate for MLG identification. Population structure analysis based on hierarchical clustering identified two subpopulations and the Bayesian approach suggested five clusters. Additionally, discriminant analysis of principal components (DAPC) separated the cassava accessions into 13 subpopulations. A comparison of these results and those of a previous study using single nucleotide polymorphisms (SNP) suggests that each type of marker can be used to assess the genetic structure of cassava grown in Burkina Faso.

## 1. Introduction

Cassava (*Manihot esculenta* Crantz, Family: Euphorbiaceae) is an important root crop, widely cultivated in Africa [1] for its tuberous roots rich in starch [2,3] and its leaves rich in protein, minerals, vitamins and carotenoids [4]. It is an important food security crop, particularly to smallholder farmers in Africa [5]. In 2021, the production of cassava in Africa was estimated as 203.57 million tons, representing 64.67% of the world’s production [6]. The crop is increasingly gaining in popularity due to its capacity to give better yields than most of the crops in the drought-prone ecologies and in poor soils [7] and its flexibility in planting and harvesting times [8].

Cassava is an allogamous species [9]. In traditional farming systems, the coexistence of different cassava accessions in the same or neighboring fields is common. This coexistence leads, thanks to cross-pollination, to an increase in genetic diversity in fields [10]. In addition, the presence of a high diversity of accessions in the fields due to the exchange of planting materials between farmers is very frequent [11]. As a result, depending on the collection localities, different accessions may have the same name, while an accession could be given different names. This leads to the presence of duplicates among accessions collected in different localities [12]. The ability to identify and remove duplicates from a collected germplasm is very important for breeding activities. In addition, the success in a breeding program depends on a good understanding of the genetic variability within the existing population. Therefore, it is important to carry out studies to identify duplicated accessions and assess the genetic diversity within accessions in order to provide breeding programs with unique genotypes [1,13]. An assessment of cassava genetic diversity has been carried out using morphological descriptors [14,15] and molecular markers [1,16,17,18]. However, morphological descriptors are known to be affected by the interaction between genotype and environment. On the other hand, the molecular markers are stable, easily detectable, and not influenced by the environment [19,20]. Various molecular tools can be used to assess the genetic diversity of crops, including Random Amplified Polymorphic DNA (RAPD) [21], Restriction Fragment Length Polymorphism (RFLP) [21], Amplified Fragment Length Polymorphism (AFLP) [22], simple sequence repeat (SSR) [1,19,23], single nucleotide polymorphism (SNP) [13,18,24,25,26] and Diversity Arrays Technology (DArT) [27,28]. The locus-specific markers such as SSR markers have found their preferential application in genetic diversity and population structure assessment in many crops [1,29,30,31]. With the possibility of whole-genome sequencing and of detecting single nucleotide polymorphisms (SNPs), SNPs have also gained in importance in genetic diversity and population structure studies [9,13,18,24].

Genomic analysis and the identification of potential duplicate accessions in cassava germplasms based on SNPs have been conducted in Burkina Faso. A high rate of potential duplicates (52.41%) and a complex genetic structure of accessions were observed [32]. The polymorphisms of SSRs and SNPs are generated via different mechanisms and the two types of markers can therefore provide different views of the diversity of a given population [33]. A total of 132 accessions were selected from the Burkina Faso cassava germplasm and genotyped using SSR markers in order to estimate the genetic diversity and the number of unique multilocus genotypes.

## 2. Materials and Methods

### 2.1. Plant Material

A total of 132 accessions (Table S1) were randomly selected from Burkina Faso cassava germplasm [32], among which 125 accessions came from seven major cassava-growing regions of Burkina Faso (Est, Centre-Ouest, Centre-Sud, Sud-Ouest, Cascades, Centre-Est and Hauts-Bassins), while seven (07) varieties originated from the International Institute of Tropical Agriculture (IITA). In the rest of this document, varieties are considered as accessions. A cutting of 20 cm of each accession was grown in a pot as described in the previous study [32]. After one month, the fully expanded leaves from each cassava accession were collected, placed into envelopes and oven-dried at 37 °C for 72 h before molecular analysis.

### 2.2. DNA Extraction

Total DNA of each sample was extracted from cassava leaves using the CTAB protocol as previously described [34]. About 30 mg of the dried leaves from each accession was ground in 2 mL Eppendorf tubes into fine powder using a TissueLyser II ball mill (Qiagen, Paris, France). Then, 800 μL of the extraction buffer (2% cetyltrimethylammonium bromide, 1.4 M of NaCl, 0.5 M of glucose, 20 mM of ethylene diamine tetra-acetic acid, 100 mM of Tris-HCl, pH 8.0) was added. The powder and the extraction buffer were shaken well in order to obtain a homogeneous mixture using a shaker (Vortex Genie^®^ 2, Scientific Industries, Bohemia, NY, USA). The sample was incubated in a water bath at 60 °C with gentle agitation for 60 min. The sample was removed from the water bath and an equal volume (800 μL) of chloroform isoamyl alcohol (24:1) was added to the supernatant previously collected into 2 mL tubes. The tube was inverted several times to ensure that a thorough mixture was obtained and then centrifuged at 20,000× *g* for 10 min at 4 °C. After centrifugation, the supernatant was transferred into a clean 1.5 mL tube and a two-thirds volume of ice-cold isopropanol (480 μL) was added whilst shaking gently for nucleic acid precipitation. Precipitation was enhanced by storing the samples at −20 °C for 30 min. Pelleting of nucleic acids was carried out by centrifuging at 20,000× *g* for 10 min. The isopropanol was removed, and the pellet was washed with 500 μL of 70% ethanol. After washing, centrifugation was carried out at 20,000× *g* for 5 min. The ethanol was decanted, and the pellet was dried. The DNA was suspended in 100 μL of molecular-biology-grade water. The quality and concentration of each DNA sample were determined using a Nanodrop 2000 spectrophotometer (Thermo Fisher Scientific, Waltham, MA, USA). DNA concentration was adjusted to 20 ng/μL.

### 2.3. SSR Marker Genotyping

#### 2.3.1. SSR Markers and Polymerase Chain Reaction (PCR)

A total of 37 simple sequence repeat (SSR) primers were used for this study (Table 1). These markers were selected based on their polymorphic profile, reproducible allele patterns, high polymorphic information content (PIC) and wide distribution across the cassava genome according to several authors [1,35,36,37,38]. Polymerase chain reactions were carried out at Laboratoire de Virologie et de Biotechnologie Végétale (Burkina Faso) using a SimpliAmp thermal cycler (Life Technologies Holdings Pte Ltd., Singapore). The PCR mix was prepared in a final volume 10 µL (1.0 µL of buffer (10×), 0.2 µL of dNTPs (10 mM), 0.2 µL of both forward and reverse primer (10 µM), 0.04 µL of 5 U/µL of Maximo Taq DNA polymerase (GeneON, San Antonio, TX, USA) and 1.0 µL of genomic DNA template (20 ng/µL) all together with 7.36 µL of molecular-biology-grade water). PCR amplifications were carried out with the following conditions: initial denaturation at 94 °C for 2 min, denaturing at 94 °C for 30 s, annealing X °C (depending on primers, Table 1) for 1 min and extension at 72 °C for 1 min. The reaction was repeated for 30 cycles and a final extension at 72 °C for 5 min was carried out. The reactions were then held at 4 °C until electrophoresis.

#### 2.3.2. Gel Electrophoresis

Gel electrophoresis was carried out according to Kirkhouse Trust Horizontal PAGE Protocol (https://www.kirkhoustrust.org/files/ugd/b134c0_edb8b37b14b14bb2a2c19e73b6651786.pdf, URL accessed on 25 November 2023). After PCR, amplified DNA fragments were separated on 6% polyacrylamide gel (16.5 mL of acrylamide bis-acrylamide (19:1), 1.1 mL of Tris-acetate-EDTA (TAE, 50×), 1.8 mL of ammonium persulfate (10%) and 91.7 µL of Tetramethyl ethylenediamine (TEMED) all together with 90.5 mL of distilled water). Electrophoresis was carried out in 0.5× TAE running buffer at 200 V for 2 h using 4.5 μL of the amplified PCR products. Then, a 100 bp DNA ladder (Solis Biodyne, Tartu, Estonia) was used to estimate the molecular weight of the amplified products. After electrophoresis, the gel was soaked in a 0.5 μg/mL ethidium bromide solution for 10 min. The PCR products were visualized and photographed using a Compact Digimage System, UVDI series (MS major science).

#### 2.3.3. Band Scoring

The SSR amplified bands were scored as diploid and codominant by visual inspection. When a single band was observed at locus *x* for a given accession, the accession was identified at this locus as homozygous. On the other hand, when two separate bands were scored at locus *x* for a given accession, the accession was represented as heterozygous at this locus.

### 2.4. Analysis of Genetic Diversity

The minimum number of markers that should be used to properly assess the genetic diversity of plant species depends on the type of marker and the genetic diversity within the species [39]. However, regardless of the type of marker or the genetic diversity of the species, it is important to identify the minimum number of markers for which the diversity within the population will not change if additional markers are added [40]. To determine this minimum number of markers needed to assess the genetic diversity of cassava accessions, a genotype accumulation curve was performed using the function *genotype_curve* in the package *poppr* as implemented in R v. 4.0.2. Prior to performing the genotype accumulation curve, SSR markers and cassava accessions with more than 6% missing data were removed from dataset [13]. The retained markers were subjected to genetic diversity analyses. Polymorphic information content (PIC), major allele frequency (MaF), observed heterozygosity (H_o_), expected heterozygosity (H_e_) and allele number per locus (AnL) were obtained using PowerMarker v. 3.2.5 [41], while Wright’s F-statistics were calculated using the package *hierfstat* [42].

### 2.5. Analysis of Genetic Structure

A principal coordinate analysis (PCoA) was performed in order to reveal the genetic relationships among the cassava accessions. This analysis was carried out using the package *cmdscale* [43]. The graphs were generated using the function *ggplot* in the package *ggplot2* [44]. All packages are implemented in R v. 4.0.2.

The function *hclust* in the package *stats* was used to build a Ward’s minimum variance hierarchical clustering dendrogram. The optimal number of clusters was evaluated using the function *best.cutree* in the package *JLutils* [45] under the assumption that the number of clusters was between 1 and 20. The duplicate accessions were identified on the basis of genetic distances between two representatives of the same accession. A threshold of 0.05 was defined as the minimum distance for considering two genotypes to be different. The duplicate accessions were also identified based on the detection of unique multilocus genotypes (MLGs) using the function *mlg.id* in the package *poppr*. The same threshold was used.

The population structure of cassava accessions was analyzed using the Bayesian approach. The clustering algorithm based on the ADMIXTURE model, implemented in STRUCTURE v. 2.3.4 [46], was used. The most likely number of clusters (k) was deduced using 15 independent iterations for each value of k (ranging from 1 to 20), with 50,000 run-in steps followed by 500,000 Markov Chain Monte Carlo (MCMC) simulations. The best value of k (Δk) was determined according to the method described by Evanno et al. [47] using STRUCTURE HARVESTER [48]. The probability matrix Q from the analysis was used to assign accessions to their groups. Accessions with a membership probability (Q) ≥80% were assigned to a cluster, while those with a membership probability below 80% were considered as a mixture (ADMIXTURE).

The package *adegenet* [49] implemented in R v. 4.0.2. was used to perform the discriminant analysis of principal components (DAPC). The best number of clusters was assessed using the function *find.clusters* implemented in the package *adegenet*. The lowest BIC value is assigned to represent the most probable number of clusters. DAPC was performed as described previously [32].

Analysis of molecular variance (AMOVA) was performed using the function *poppr.amova* in the package *poppr*. The principal components were decomposed into different hierarchical levels: breeding patterns, geographical origin and the theoretical clusters obtained by Bayesian approach and by DAPC.

## 3. Results

### 3.1. Genetic Diversity Parameters

**Figure 1:**
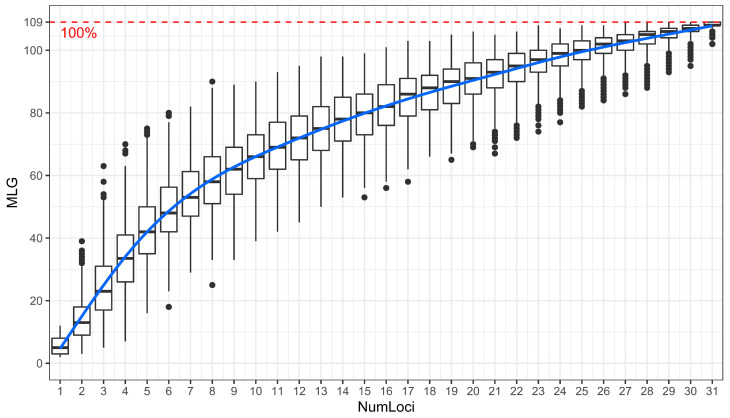
Genotype accumulation curve obtained using 32 SSR markers.

All the SSR markers were amplified with less than 6% of missing data except for marker SSRY127. The markers SSRY132, SSRY171 and SSRY181 generated one allele/locus. The 4 markers and 2 accessions, with more than 6% of missing data, were removed from the initial dataset, leaving a final dataset consisting of 32 SSR markers and 130 accessions. The genotype accumulation curves obtained, using this dataset, showed that the data reached a small plateau and had a greatly decreased variance with 31 SSR markers, indicating that there were enough markers for the accession’s discrimination. This curve revealed the presence of 109 (83.8%) unique multilocus genotypes (Figure 1).

The diversity parameters estimated are reported in Table 2. The 32 SSR markers generated a total of 105 alleles ranging from 2 to 6 per locus, with an average of 3.3. The SSRY20 recorded the highest number of 6 alleles per locus. The PIC values ranged from 0.03 to 0.69 with an average of 0.40. Among the 32 SSR markers, 12 were highly informative, with PIC values greater than 0.5; 12 were moderately informative, with PIC values between 0.25 and 0.50; and 8 were not very informative, with the PIC values lower than 0.25. The MaF values were from 0.62 to 0.98 with an average of 0.65. The markers SSRY38, SSRY110, SSRY161 and SSRY169 had MaF values of more than 0.95, indicating their low polymorphism. The H_e_, H_o_, F_IT_, F_IS_ and F_ST_ values estimated for accessions averaged 0.45, 0.48, −0.07, −0.10 and 0.03, respectively (Table 2).

### 3.2. Population Structure and Genetic Relationships

#### 3.2.1. Principal Coordinate Analysis (PCoA)

**Figure 2:**
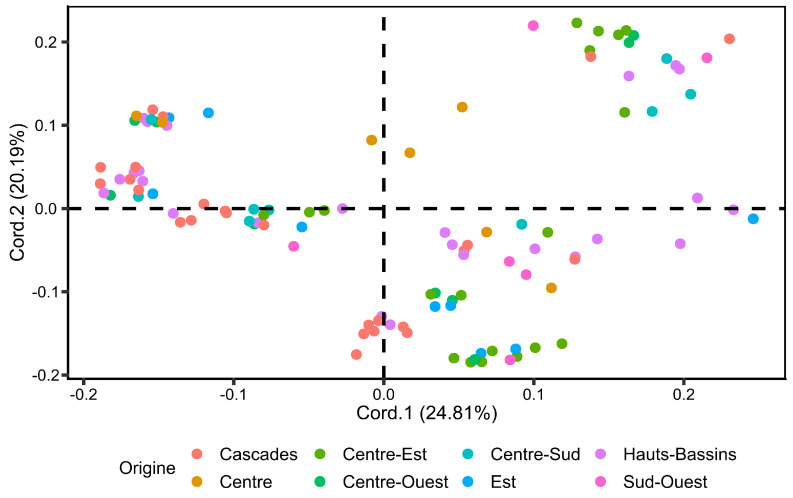
Graphical representation of principal coordinate analysis (PcoA). Accessions are colored according to geographical origin.

The principal coordinate analysis of accessions generated the graphical representations of the relationship between the accessions (Figure 2). The graphical representations was made using the first two principal coordinates (Cord.1 and Cord.2). These two coordinates accounted for 45% of the total variation. The PcoA results showed a lack of clustering of accessions according to their geographical origin.

#### 3.2.2. Hierarchical Clustering Analysis and Identification of Duplicate Accessions

**Figure 3:**
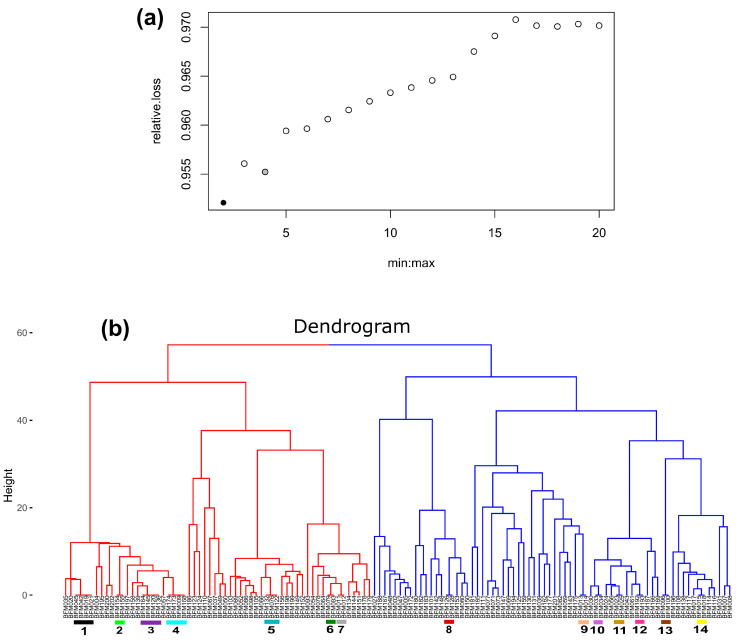
Hierarchical clustering using 32 SSR markers. (**a**) The black dot indicates the best number of clusters. (**b**) Hierarchical clustering of the 130 cassava accessions. The colored bars represent the 14 duplicate MLGs (unique multilocus genotypes) identified.

Optimal cluster number assessment and the hierarchical clustering dendrogram showed that the 130 cassava accessions could be grouped into two clusters (Figure 3a). This hierarchical clustering revealed the presence of 21 (16.2%) duplicated accessions. The duplicates belonged to 14 unique multilocus genotypes (Figure 3b). These results were confirmed by the results of duplicate identification performed using the function *mlg.id* in the package *poppr*.

#### 3.2.3. Bayesian Analysis

**Figure 4:**
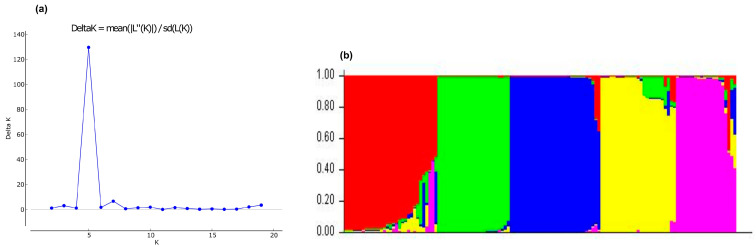
Population structure of cassava accessions according to Bayesian approach. (**a**) Plot of delta K against the number of K groups. (**b**) The colors represent the five groups based on membership probability ≥80%.

Population structure analysis based on the Bayesian approach showed that the optimal number of clusters (k) that would best explain the structure of the accessions is 5 (Figure 4a). Using a membership probability threshold of 80%, 115 accessions (88.46%) were successfully assigned to the five clusters (Figure 4b). A total of 23, 26, 28, 20 and 18 accessions were successfully assigned to cluster 1 (Q = 95%), cluster 2 (Q = 97.8%), cluster 3 (Q = 97.4%), cluster 4 (Q = 94%) and cluster 5 (Q = 98%), respectively. A total of 15 accessions (11.54%) with membership probabilities less than 80% were admixtures (Table S1).

#### 3.2.4. Discriminant Analysis of Principal Components (DAPC)

**Figure 5:**
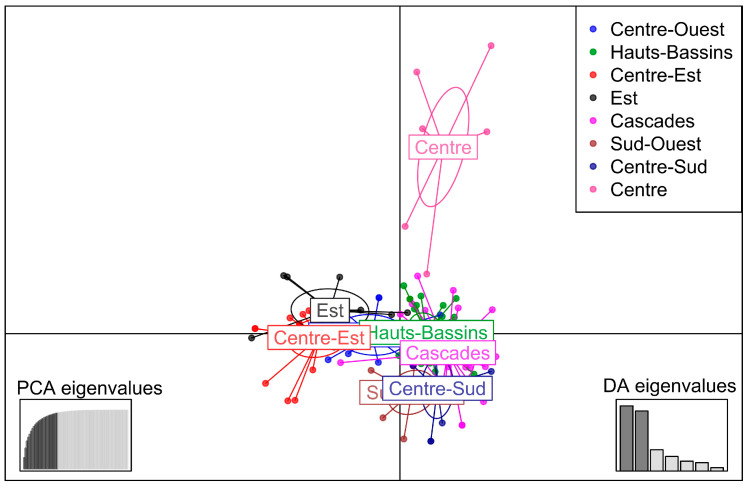
Discriminant analysis of principal components (DAPC) using the regions as predefined groups.

SSR data were used for DAPC. Primarily, this analysis was performed using the regions as predefined groups. The first 25 principal components (PCs) and eight discriminant functions were used for the DAPC. The first two discriminant functions explaining 35.7 and 32.9% of the total genetic variation, respectively, were used for the graphical representation of the DAPC results (Figure 5). Accessions were assigned to the eight regions with an average assignment probability of 65.4%. The average assignment probabilities of accessions to each region were 33.33% (Est), 58.33% (Centre-Ouest), 80% (Centre-Sud), 83.33% (Sud-Ouest), 58.62% (Cascades), 68% (Centre-Est), 71.87% (Hauts-Bassins) and 71.43% (Centre).

**Figure 6:**
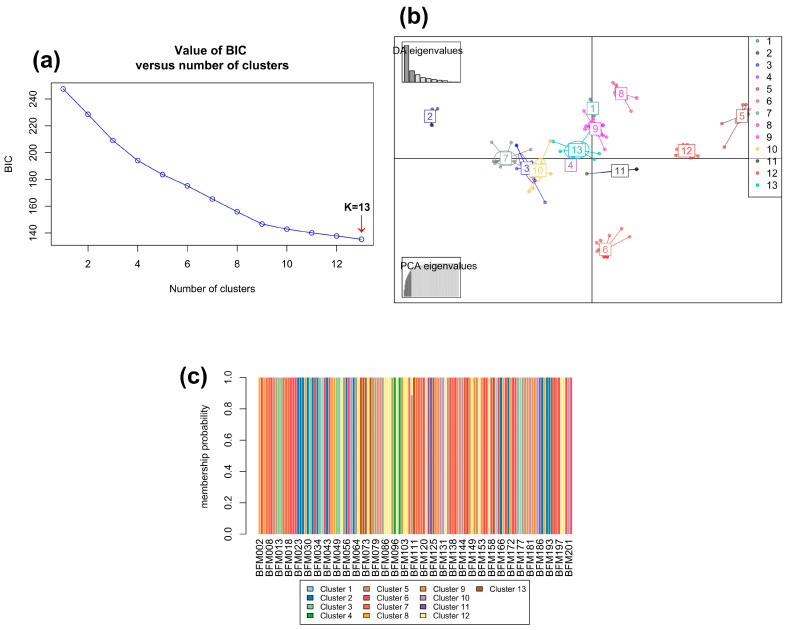
Discriminant analysis of principal components (DAPC). (**a**) Best number of clusters. (**b**) Graphical representation of the DAPC results. (**c**) Membership probability of cassava accessions.

The discriminant analysis of principal components of the 130 accessions was also performed without predefined groups. The lowest value of BIC was obtained for 13 clusters (Figure 6a). The first ten principal components (PCs) and ten discriminant functions were retained for the DAPC. The first two discriminant functions, which explained 52.1 and 16.2% of the total genetic variation, were used for the graphical representation of the DAPC results (Figure 6b). The accessions were assigned to the 13 clusters with an individual membership probability of 100% except for the BFM110 which was assigned to cluster 5 with a probability of 89% (Figure 6c).

#### 3.2.5. Analysis of Molecular Variance (AMOVA)

Analysis of molecular variance of cassava accessions based on geographical origin (regions) and breeding patterns showed that the most significant differences were within individuals (Table 3). The molecular variance within individuals based on geographical origin (regions) and breeding patterns were 93.69% and 96.67% of the total molecular variance, respectively. The AMOVA based on DAPC clusters showed that the most significant molecular variance was between groups with 70.09% (Table 3). The AMOVA, based on clusters of Bayesian analysis, revealed that the molecular variance was slightly higher within individuals (53.27%) compared to the variance between clusters (46.73%). The mean indexes of genetic differentiation of the accessions were assessed according to geographical origin, breeding patterns, DAPC clusters and Bayesian approach clusters. These results are recorded in Table 4.

## 4. Discussion

Understanding the genetic diversity of species is the basis of the success of any breeding program and leads to develop strategies for germplasm management, conservation and improvement [28]. Assessment of the genetic variability of a given population in order to provide breeding programs with interesting parental lines is a very important pre-breeding operation and must take into account the morphological and molecular variabilities in an existing population. Genetic diversity studies using morphological traits alone are limiting because of the interaction between environmental and genotype effects [50]. These limitations may not allow the accurate detection of duplicates. According to Collard et al. (2005), the use of molecular markers can permit the detection of genetic differences among closely related genotypes. In addition, assessment of the agro-morphological diversity of cassava requires a great deal of space, depending on the number of accessions, and is spread over several months (9 to 12 months) [15,28]. It is therefore advisable to assess molecular diversity within the germplasm and to identify the unique multilocus genotypes first, before assessing agro-morphological diversity.

Molecular markers need to be chosen appropriately to be ubiquitous, reasonably polymorphic, reproducible, and easily detectable [39] like SNPs and SSRs. In practice, there is no perfect molecular marker method that satisfies all expectations and does not present any challenge with its application. The choice of which marker technique to apply depends strongly on some factors such as the set objective, the level of the genetic variability of the population, the sample size, the accessibility of primers, the availability of the technical know-how and appropriate facilities, time and financial considerations [51,52]. In addition, the number of alleles depends on the type of marker. For example, SNP markers have a fixed number of alleles while SSR markers can have many alleles per locus [1]. Whatever the type of marker used, it is important to determine the minimum number of markers that can efficiently discriminate the maximal number of accessions [40].

Genomic analysis of cassava accessions and the identification of potential duplicate accessions based on SNPs conducted in Burkina Faso revealed a high rate (52.41%) of potential duplicates [32]. This high rate allowed us to genotype the accessions using SSR markers in order to estimate the genetic diversity and the number of unique multilocus genotypes (MLGs) in the Burkina Faso cassava germplasm. The 132 accessions were randomly selected from the germplasm coming from major cassava-growing regions and genotyped using 32 SSR markers. The genotype accumulation curve showed that the 32 SSR markers were sufficient for the discrimination of the 130 accessions. Moreover, it revealed the presence of 83.8% of unique multilocus genotypes (MLGs) among the population. This rate was higher than the rate of MLGs (47.6%) found in previous studies [32] despite the fewer accessions used in this study. These results indicate that the 32 SSR markers have a greater capacity to estimate the number of MLGs than the 34 SNP markers used in the previous study.

The results of the analysis of genetic diversity parameters of the 130 accessions showed that the 32 SSR markers were polymorphic with 0.40 as the mean value of PIC. This value was higher than that reported by Moyib et al. [53] but lower than those reported by other authors [1,19,54,55]. These differences could be explained by the specificity of each cassava germplasm studied and the SSR markers used. Furthermore, the mean PIC value observed in this study was higher than those observed previously in Burkina Faso using SNP markers. This difference could be explained by the bi-allelic nature of SNP markers, unlike SSR which are multi-allelic [18]. Indeed, the number of alleles per loci in this study ranged from 2 to 6. The average H_o_ in this study was higher than H_e_, suggesting a heterozygote excess within the 130 cassava accessions. This excess of heterozygotes was confirmed by negative values of the F_IS_ and F_IT_. In addition, an excess of heterozygosity in cassava populations has been reported in several studies [1,19,54,55].

Molecular profiling of accessions revealed a low rate of duplicates (16.2%) in this study compared to the previous study in which 52.41% of duplicates were found [32]. This could be explained by the few SNP markers used in the previous study (34 SNP markers). Indeed, given the multi-allelic nature of the SSR markers and the bi-allelic nature of the SNP markers, more SNP markers may be needed when compared with SSR markers to achieve the same degree of resolution [39,40]. PCoA was not able to differentiate cassava accessions according to the origin. In addition, the DAPC performed using the regions as predefined groups did not reveal a clear differentiation of accessions according to the origin. This absence of differentiation was confirmed by the low values of the genetic differentiation index (F_ST_), which was 0.025. Furthermore, the AMOVA results indicated that 93.69% of molecular variation was found within individuals with only 6.31% between regions. This could be due to the fact that some accessions are grown in several regions in Burkina Faso [32]. The analysis also revealed a weak differentiation of the accessions according to breeding patterns with a low value of F_ST_ (0.008). This absence of differentiation is probably due to the fact that most of the improved varieties are grown in cassava fields [32]. The dendrograms obtained by the hierarchical clustering showed that the 130 cassava accessions can be grouped into two large clusters. As mentioned in the previous study [32], this truncation may not reflect the real structure of the population, given that the truncation was carried out at the top of the dendrogram. The number of clusters obtained using the Bayesian approach (5 clusters) in this study was higher than that obtained in the previous study (2 clusters). That could be due to the fact the number of duplicate accessions was low in this study. Several studies argued that the low rate of duplicate accessions could improve the accuracy of the Bayesian approach [32,56]. The DAPC performed on the 130 cassava accessions divided the accessions into 13 clusters with an individual assignment probability (100%). The difference between the results of the Bayesian approach and the DAPC could be due to the multivariate approach used by the DAPC and the fact that the Bayesian approach is based on the Hardy–Weinberg equilibrium (HWE) model. However, for vegetatively propagated species such as cassava, this equilibrium is not often respected [9,56,57]. It was found that nearly 70% of molecular variance was between the clusters formed by DAPC, compared to only 30% within the accessions. In contrast, the molecular variance between clusters formed by the Bayesian approach represented 47% compared to 53% within the accessions. As a result, DAPC could be more suitable as it uses an approach that can assess genetic structures in the absence of any assumptions about the genetic model of the population [32,42]. The DAPC performed in this study suggested a number of clusters (13 clusters) fewer than that suggested by Soro et al. (17 clusters) [32]. This could be due to the number of accessions used in this study (130 accessions) being fewer than that used by Soro et al. (166 accessions). The analyses carried out on 104 accessions genotyped using SSR and SNP markers revealed the same number of clusters (10 clusters) with a higher individual assignment probability (100%) of accessions into clusters for the two types of markers (Figures S1 and S2). For both marker systems (SNP and SSR), the same number of clusters was observed by several authors by using different genetic structure assessment methods [58,59]. These results could be very useful for laboratories with limited resources. SSR markers are available for several crops and the SSR genotyping technique can be implemented in any molecular biology laboratory.

## 5. Conclusions

In the present study, we explored the genetic diversity and relationships within and between cassava accessions collected in cassava-growing regions of Burkina using SSR markers. The analysis of genetic diversity parameters indicated significant genetic diversity in the cassava accessions. The population structure suggested by DAPC was more efficient than that suggested by the Bayesian approach. However, the analyses revealed that SSR markers seem to be the most appropriate for MLG identification. In addition to the assessment of genetic diversity, we plan to assess the agro-morphological diversity and disease resistance status of MLGs in order to establish a national cassava germplasm bank, which would be very useful for breeding programs.

## Figures and Tables

**Table 1:** Characteristics of simple sequence repeat (SSR) primers used for this study.

Markers	Forward Primer	Reverse Primer	PS (pb)	AT (°C)
SSRY4	ATAGAGCAGAAGTGCAGGCG	CTAACGCACACGACTACGGA	287	55
SSRY9	ACAATTCATCATGAGTCATCAACT	CCGTTATTGTTCCTGGTCCT	278	55
SSRY12	AACTGTCAAACCATTCTACTTGC	GCCAGCAAGGTTTGCTACAT	266	55
SSRY19	TGTAAGGCATTCCAAGAATTATCA	TCTCCTGTGAAAAGTGCATGA	214	55
SSRY20	CATTGGACTTCCTACAAATATGAAT	TGATGGAAAGTGGTTATGTCCTT	143	55
SSRY21	CCTGCCACAATATTGAAATGG	CAACAATTGGACTAAGCAGCA	192	55
SSRY34	TTCCAGACCTGTTCCACCAT	ATTGCAGGGATTATTGCTCG	279	55
SSRY38	GGCTGTTCGTGATCCTTATTAAC	GTAGTTGAGAAAACTTTGCATGAG	122	55
SSRY51	AGGTTGGATGCTTGAAGGAA	GGATGCAGGAGTGCTCAACT	298	55
SSRY59	GCAATGCAGTGAACCATCTTT	CGTTTGTCCTTTCTGATGTTC	158	55
SSRY63	TCAGAATCATCTACCTTGGCA	AAGACAATCATTTTGTGCTCCA	290	55
SSRY64	CGACAAGTCGTATATGTAGTATTC	GCAGAGGTGGCTAACGAGAC	194	55
SSRY69	CGATCTCAGTCGATACCCAAG	CACTCCGTTGCAGGCATTA	239	55
SSRY82	TGTGACAATTTTCAGATAGCTTCA	CACCATCGGCATTAAACTTTG	211	55
SSRY100	ATCCTTGCCTGACATTTTGC	TTCGCAGAGTCCAATTGTTG	210	55
SSRY102	TTGGCTGCTTTCACTAATGC	TTGAACACGTTGAACAACCA	179	55
SSRY103	TGAGAAGGAAACTGCTTGCAC	CAGCAAGACCATCACCAGTTT	272	55
SSRY105	CAAACATCTGCACTTTTGGC	TCGAGTGGCTTCTGGTCTTC	225	55
SSRY106	GGAAACTGCTTGCACAAAGA	CAGCAAGACCATCACCAGTTT	270	55
SSRY108	ACGCTATGATGTCCAAAGGC	CATGCCACATAGTTCGTGCT	203	55
SSRY110	TTGAGTGGTGAATGCGAAAG	AGTGCCACCTTGAAAGAGCA	247	55
SSRY127	GCTGAACTGCTTTGCCAACT	CTTCGGCCTCTACAAAAGGA	130	45
SSRY132	CTTTTTGCCAGTCTTCCTGC	TGTCCAATGTCTTCCTTTCCTT	196	55
SSRY135	CCAGAAACTGAAATGCATCG	AACATGTGCGACAGTGATTG	253	45
SSRY147	GTACATCACCACCAACGGGC	AGAGCGGTGGGGCGAAGAGC	113	55
SSRY148	GGCTTCATCATGGAAAAACC	CAATGCTTTACGGAAGAGCC	114	55
SSRY151	AGTGGAAATAAGCCATGTGATG	CCCATAATTGATGCCAGGTT	182	55
SSRY155	CGTTGATAAAGTGGAAAGAGCA	ACTCCACTCCCGATGCTCGC	158	55
SSRY161	AAGGAACACCTCTCCTAGAATCA	CCAGCTGTATGTTGAGTGAGC	220	55
SSRY169	TCAAACAAGAATTAGCAGAACTGG	TGAGATTTCGTAATATTCATTTCACTT	187	45
SSRY171	ACAGCTCTAAAAACTGCAGCC	AACGTAGGCCCTAACTAACCC	100	55
SSRY177	ACTGTGCCAAAATAGCCAAATAGT	TCATGAGTGTGGGATGTTTTTATG	291	55
SSRY179	ACCACAAACATAGGCACGAG	CACCCAATTCACCAATTACCA	268	45
SSRY180	CAGGCTCAGGTGAAGTAAAGG	GCGAAAGTAAGTCTACAACTTTTCTAA	226	55
SSRY181	CCTTGGCAGAGATGAATTAGAG	GGGGCATTCTACATGATCAATAA	163	55
SSRY182	GGTAGATCTGGATCGAGGAGG	CAATCGAAACCGACGATACA	199	55

PS = product size; AT = annealing temperature.

**Table 2:** Common genetic parameters and F-statistics for each SSR locus.

Markers	MaF	AnL	H_e_	H_o_	F_IT_	F_IS_	F_ST_	PIC
SSRY4	0.35	5	0.74	0.65	0.05	0.03	0.02	0.69
SSRY9	0.67	4	0.51	0.53	−0.01	−0.05	0.04	0.48
SSRY12	0.51	3	0.58	0.98	−0.70	−0.74	0.02	0.49
SSRY19	0.58	4	0.58	0.75	−0.24	−0.31	0.05	0.51
SSRY20	0.50	6	0.68	0.77	−0.16	−0.20	0.04	0.65
SSRY21	0.72	4	0.43	0.54	−0.25	−0.28	0.02	0.39
SSRY34	0.94	2	0.12	0.11	0.20	0.19	0.01	0.11
SSRY38	0.98	3	0.03	0.03	−0.02	−0.08	0.06	0.03
SSRY51	0.38	3	0.66	0.90	−0.35	−0.35	0.00	0.59
SSRY59	0.46	4	0.62	0.98	−0.57	−0.58	0.01	0.54
SSRY63	0.60	3	0.55	0.81	−0.46	−0.50	0.02	0.48
SSRY64	0.70	2	0.42	0.25	0.39	0.33	0.09	0.33
SSRY69	0.50	5	0.68	0.62	0.13	0.12	0.01	0.64
SSRY82	0.40	4	0.68	0.78	−0.12	−0.15	0.03	0.62
SSRY100	0.36	5	0.69	0.63	0.02	0.02	0.00	0.63
SSRY102	0.78	2	0.35	0.45	−0.26	−0.26	0.00	0.29
SSRY103	0.58	3	0.57	0.60	−0.03	−0.05	0.01	0.51
SSRY105	0.93	3	0.14	0.13	0.18	0.16	0.03	0.13
SSRY106	0.72	3	0.43	0.47	−0.11	−0.15	0.04	0.37
SSRY108	0.76	4	0.39	0.48	−0.23	−0.22	−0.01	0.36
SSRY110	0.97	2	0.06	0.06	−0.02	−0.02	0.00	0.06
SSRY135	0.69	3	0.47	0.40	0.09	0.04	0.05	0.43
SSRY147	0.61	2	0.48	0.66	−0.36	−0.37	0.01	0.36
SSRY148	0.95	2	0.09	0.09	−0.05	−0.09	0.03	0.08
SSRY151	0.32	5	0.73	0.87	−0.17	−0.16	−0.01	0.68
SSRY155	0.58	2	0.49	0.26	0.47	0.45	0.02	0.37
SSRY161	0.97	2	0.06	0.06	−0.03	−0.04	0.01	0.06
SSRY169	0.96	2	0.08	0.08	−0.04	−0.06	0.02	0.08
SSRY177	0.47	4	0.67	0.46	0.28	0.27	0.00	0.61
SSRY179	0.63	3	0.52	0.21	0.47	0.43	0.06	0.44
SSRY180	0.84	2	0.27	0.28	−0.13	−0.17	0.03	0.23
SSRY182	0.45	4	0.65	0.53	0.17	0.12	0.06	0.58
Mean	0.65	3.3	0.45	0.48	−0.07	−0.10	0.03	0.40

MaF = major allele frequency; AnL = allele number per locus; H_e_ = expected heterozygosity; H_o_ = observed heterozygosity; F_IT_ = inbreeding coefficient of an individual into the whole population; F_IS_ = within-population inbreeding coefficient; F_ST_ = coefficient of differentiation; and PIC = polymorphic information content.

**Table 3:** AMOVA of the 130 accessions performed considering geographical origin, breeding patterns and theoretical clusters obtained by Bayesian approach and by DAPC.

**Source of Variation**	**Geographical Origin**			**Source of Variation**	**Breeding Patterns**		
	**df**	**Mean Sq**	**% of Variation**		**df**	**Mean Sq**	**% of Variation**
Between clusters	7	13.04	6.31	Between groups	1	14.83	3.33
Within individuals	122	6.40	93.69	Within individuals	128	6.70	96.67
Total	129	6.76	100.00	Total	129	6.78	100.00
**Source of Variation**	**DAPC**			**Source of Variation**	**Bayesian Approach**		
	**Df**	**Mean Sq**	**% of Variation**		**df**	**Mean Sq**	**% of Variation**
Between groups	12	51.55	70.09	Between groups	4	94.06	46.73
Within individuals	117	2.17	29.91	Within individuals	125	3.97	53.27
Total	129	6.76	100.00	Total	129	6.76	100.00

**Table 4:** F_ST_ of the 130 accessions according to geographical origin, breeding patterns, DAPC clusters and Bayesian clusters.

SNP Markers	
Type of Clustering	F_ST_
Geographical origin	0.025
Breeding patterns	0.008
DAPC clusters	0.307
Bayesian clusters	0.192

## Data Availability

The accession number(s) can be found through this link: https://www.ebi.ac.uk/biostudies/studies/S-BSST1233?key=314eafaa-d6d5-4cf1-bda2-81203bf2670d.

## Supplementary Materials

The following supporting information can be downloaded at https://www.mdpi.com/article/10.3390/genes15010073/s1. Table S1: Classification of the 130 cassava accessions into groups based on geographical origin, DAPC analysis and Bayesian approach using 32 SSR markers. Figure S1: Discriminant analysis of principal components (DAPC) of 104 cassava accessions obtained from the analysis of 34 SNP markers. (a) Optimal number of clusters. (b) Graphical representation of the DAPC results. Clusters are represented by colors according to the legend. (c) Membership probability of cassava accessions. Each accession is represented by a vertical line and the colors correspond to the probability of assignment in each of the 10 groups. Figure S2: Discriminant analysis of principal components (DAPC) of 104 cassava accessions obtained from the analysis of 32 SSR markers. (a) Optimal number of clusters. (b) Graphical representation of the DAPC results. Clusters are represented by colors according to the legend. (c) Membership probability of cassava accessions. Each accession is represented by a vertical line and the colors correspond to the probability of assignment in each of the 10 groups.

## References

[1] Adjebeng-Danquah J., Manu-Aduening J., Asante I.K., Agyare R.Y., Gracen V., Offei S.K. (2020). Genetic diversity and population structure analysis of Ghanaian and exotic cassava accessions using simple sequence repeat (SSR) markers. Heliyon.

[2] Montagnac J.A., Davis C.R., Tanumihardjo S.A. (2009). Nutritional value of cassava for use as a staple food and recent advances for improvement. Compr. Rev. Food Sci. Food Saf..

[3] Salvador E.M., Steenkamp V., McCrindle C.M.E. (2014). Production, consumption and nutritional value of cassava (*Manihot esculenta*, Crantz) in Mozambique: An overview. J. Agric. Biotechnol. Sustain. Dev..

[4] Latif S., Müller J. (2015). Potential of cassava leaves in human nutrition: A review. Trends Food Sci. Technol..

[5] Ally H.M., Hamss H., Simiand C., Maruthi M.N., Colvin J., Omongo C.A., Delatte H. (2019). What has changed in the outbreaking populations of the severe crop pest whitefly species in cassava in two decades?. Sci. Rep..

[6] FAOSTAT Food and Agriculture Organization of the United Nations Statistics Division. https://www.fao.org/faostat/en/#data/QCL.

[7] El-Sharkawy M.A. (2007). Physiological characteristics of cassava tolerance to prolonged drought in the tropics: Implications for breeding cultivars adapted to seasonally dry and semiarid environments. Brazilian J. Plant Physiol..

[8] El-Sharkawy M.A. (2004). Cassava biology and physiology. Plant Mol. Biol..

[9] de Oliveira E.J., Ferreira C.F., Silva S.D.V., Jesus O.D.N., Oliveira G.A.F., Silva M.D.S. (2014). Potential of SNP markers for the characterization of Brazilian cassava germplasm. Theor. Appl. Genet.

[10] Elias M., Penet L., Vindry P., McKey D., Panaud O., Robert T. (2001). Unmanaged sexual reproduction and the dynamics of genetic diversity of a vegetatively propagated crop plant, cassava (*Manihot esculenta* Crantz), in a traditional farming system. Mol. Ecol..

[11] Park Y., Dixit A., Ma K., Kang J., Rao V.R., Cho E. (2005). On-farm Conservation Strategy to Ensure Crop Genetic Diversity in Changing Agro-ecosystems in the Republic of Korea. J. Agron. Crop Sci..

[12] Rao S.A., Bounphanousay C., Schiller J.M., Alcantara A.P., Jackson M.T. (2002). Naming of traditional rice varieties by farmers in the Lao PDR. Genet. Resour. Crop Evol..

[13] Ferguson M.E., Shah T., Kulakow P., Ceballos H. (2019). A global overview of cassava genetic diversity. PLoS ONE.

[14] Kawuki R.S., Ferguson M., Labuschagne M.T., Herselman L., Orone J., Ralimanana I., Bidiaka M., Lukombo S., Kanyange M.C., Gashaka G. (2011). Variation in qualitative and quantitative traits of cassava germplasm from selected national breeding programmes in sub-Saharan Africa. Field Crops Res..

[15] Kamanda I., Blay E.T., Asante I.K., Danquah A., Ifie B.E., Parkes E., Kulakow P., Rabbi I., Conteh A., Kamara J.S. (2020). Genetic diversity of provitamin-A cassava (*Manihot esculenta* Crantz) in Sierra Leone. Genet. Resour. Crop Evol..

[16] Gonçalves T.M., Filho P.S.V., Vidigal M.C.G., Ferreira R.C.U., Rocha V.P.C., Ortiz A.H.T., Moiana L.D., Kvitschal M.V. (2017). Genetic diversity and population structure of traditional sweet cassava accessions from Southern of Minas Gerais State, Brazil, using microsatellite markers. Afr. J. Biotechnol..

[17] Albuquerque H.D.Y.G., Oliveira E.D.J., Brito A.C., Andrade L.D.R.B., Carmo C.D.D., Morgante C.V., Vieira E.A., Moura E.F., Faleiro F.G. (2019). Identification of duplicates in cassava germplasm banks based on single-nucleotide polymorphisms (SNPs). Sci. Agric..

[18] Prempeh R.N.A., Manu-Aduening J.A., Quain M.D., Asante I.K., Offei S.K., Danquah E.Y. (2020). Assessment of genetic diversity among cassava landraces using single nucleotide polymorphic markers. Afr. J. Biotechnol..

[19] Asare P.A., Galyuon I.K.A., Sarfo J.K., Tetteh J.P. (2011). Morphological and molecular based diversity studies of some cassava (*Manihot esculenta* crantz) germplasm in Ghana. Afr. J. Biotechnol..

[20] Mezette T.F., Blumer C.G., Veasey E.A. (2013). Morphological and molecular diversity among cassava genotypes. Pesqui. Agropecuária Bras..

[21] Fregene M., Angel F., Gómez R., Rodriguez F., Chavarriaga P., Roca W., Tohme J., Bonierbale M. (1997). A molecular genetic map of cassava (*Manihot esculenta* Crantz). Theor. Appl. Genet..

[22] Elias M., Panaud O., Robert T. (2000). Assessment of genetic variability in a traditional cassava (*Manihot esculenta* Crantz) farming system, using AFLP markers. Heredity.

[23] Kawuki R.S., Herselman L., Labuschagne M.T., Nzuki I., Ralimanana I., Bidiaka M., Kanyange M.C., Gashaka G., Masumba E., Mkamilo G. (2013). Genetic diversity of cassava (*Manihot esculenta* Crantz) landraces and cultivars from southern, eastern and central Africa. Plant Genet. Resour..

[24] Albuquerque H.D.Y.G., Carmo C.D.D., Brito A.C., Oliveira E.D.J. (2018). Genetic diversity of Manihot esculenta Crantz germplasm based on single-nucleotide polymorphism markers. Ann. Appl. Biol..

[25] Adu B.G., Yeboah A., Akromah R., Bobobee E., Amoah S., Kena A.W., Amoah R.A. (2020). Whole genome SNPs and phenotypic characterization of cassava (*Manihot esculenta* Crantz) germplasm in the semi-deciduous forest ecology of Ghana. Ecol. Genet. Genom..

[26] Karim K.Y., Ifie B., Dzidzienyo D., Danquah E.Y., Blay E.T., Whyte J.B.A., Kulakow P., Rabbi I., Parkes E., Omoigui L. (2020). Genetic characterization of cassava (*Manihot esculenta* Crantz) genotypes using agro-morphological and single nucleotide polymorphism markers. Physiol. Mol. Biol. Plants.

[27] Nelimor C., Badu-Apraku B., Garcia-Oliveira A.L., Tetteh A., Paterne A., N’guetta A.S.-P., Gedil M. (2020). Genomic Analysis of Selected Maize Landraces from Sahel and Coastal West Africa Reveals Their. Genes.

[28] Pierre N., Wamalwa L.N., Muiru W.M., Simon B., Kanju E., Ferguson M.E., Ndavi M.M., Tumwegamire S. (2022). Genetic diversity of local and introduced cassava germplasm in Burundi using DArTseq molecular analyses. PLoS ONE.

[29] Li X., Qiao L., Chen B., Zheng Y., Zhi C., Zhang S., Pan Y. (2022). Plant Diversity SSR markers development and their application in genetic diversity evaluation of garlic (*Allium sativum*) germplasm. Plant Divers..

[30] Luo Z., Yao Z., Yang Y., Wang Z., Zou H., Zhang X., Chen J. (2023). Genetic fingerprint construction and genetic diversity analysis of sweet potato (*Ipomoea batatas*) germplasm resources. BMC Plant Biol..

[31] Suvi W.T., Shimelis H., Laing M., Mathew I., Titus W., Shimelis H., Laing M., Mathew I. (2020). Assessment of the genetic diversity and population structure of rice genotypes using SSR markers. Acta Agric. Scand. Sect. B—Soil. Plant Sci..

[32] Soro M., Pita J.S., Somé K., Otron D.H., Yéo E., Mutuku J.M., Néya J.B., Tiendrébéogo F., Koné D. (2023). Genomic analysis and identification of potential duplicate accessions in Burkina Faso cassava germplasm based on single nucleotide polymorphism. Front. Sustain. Food Syst..

[33] Singh N., Choudhury D.R., Singh A.K., Kumar S., Srinivasan K., Tyagi R.K., Singh N.K., Singh R. (2013). Comparison of SSR and SNP Markers in Estimation of Genetic Diversity and Population Structure of Indian Rice Varieties. PLoS ONE.

[34] Permingeat H.R., Romagnoli M.V., Juliana I., Vallejos R.H. (1998). A Simple Method for Isolating DNA of High Yield and Quality from Cotton (*Gossypium hirsutum* L.) Leaves. Plant Mol. Biol. Report.

[35] Mba R.E.C., Stephenson P., Edwards K., Melzer S., Nkumbira J., Gullberg U., Apel K., Gale M., Tohme J., Fregene M. (2001). Simple sequence repeat (SSR) markers survey of the cassava (*Manihot esculenta* Crantz) genome: Towards an SSR-based molecular genetic map of cassava. Theor. Appl. Genet..

[36] Twumasi P., Acquah E.W., Quain M.D., Parkes E.Y. (2014). Use of simple sequence repeat (SSR) markers to establish genetic relationships among cassava cultivars released by different research groups in Ghanaian. Int. J. Genet. Mol. Biol..

[37] Beovides Y., Fregene M., Gutiérrez J.P., Milián M.D., Coto O., Buitrago C., Cruz J.A., Ruiz E., Basail M., Rayas A. (2015). Molecular diversity of Cuban cassava (*Manihot esculenta* Crantz) cultivars assessed by simple sequences repeats (SSR). Biotechnol. Agron. Soc. Environ..

[38] Acquah W.E., Quain D.M., Twumasi P. (2011). Genetic relationships between some released and elite Ghanaian cassava cultivars based on distance matrices. Afr. J. Biotechnol..

[39] Grünwald N.J., Everhart S.E., Kamvar Z.N. (2017). Best Practices for Population Genetic Analyses. Phytopathology.

[40] Arnaud-Haond S., Duarte C.M., Alberto F., Serrao E.A. (2007). Standardizing methods to address clonality in population studies. Mol. Ecol..

[41] Liu K., Muse S.V. (2005). PowerMaker: An integrated analysis environment for genetic maker analysis. Bioinforma Appl. Note.

[42] Goudet J., Jombart T., Kamvar Z.N., Archer E., Hardy O. (2020). Estimation and Tests of Hierarchical F-Statistics. https://cran.r-project.org/web/packages/hierfstat/hierfstat.pdf.

[43] Oksanen J., Blanchet F.G., Friendly M., Kindt R., Legendre P., McGlinn D., Minchin P.R., O’Hara R.B., Simpson G.L., Solymos P. (2020). Vegan: Community Ecology Package.

[44] Villanueva R.A.M., Chen Z.J. (2019). ggplot2: Elegant Graphics for Data Analysis (2nd ed.). Meas. Interdiscip. Res. Perspect..

[45] Larmarange J. (2021). JLutils: Collection of R Functions. https://github.com/larmarange/JLutils.

[46] Pritchard J.K., Stephens M., Donnelly P. (2000). Inference of Population Structure Using Multilocus Genotype Data. Genetics.

[47] Evanno G., Regnaut S., Goudet J. (2005). Detecting the number of clusters of individuals using the software STRUCTURE: A simulation study. Mol. Ecol..

[48] Earl D.A., vonHoldt B.M. (2012). STRUCTURE HARVESTER: A website and program for visualizing STRUCTURE output and implementing the Evanno method. Conserv. Genet. Resour..

[49] Jombart T., Devillard S., Balloux F. (2010). Discriminant analysis of principal components: A new method for the analysis of genetically structured populations. BMC Genet..

[50] Collard B.C.Y., Jahufer M.Z.Z., Brouwer J.B., Pang E.C.K. (2005). An introduction to markers, quantitative trait loci (QTL) mapping and marker-assisted selection for crop improvement: The basic concepts. Euphytica.

[51] Anne C. (2006). Choosing the right molecular genetic markers for studying biodiversity: From molecular evolution to practical aspects. Genetica.

[52] Amiteye S. (2021). Basic Concepts And Methodologies Of DAN Marker Systems in Plant Molecular Breeding. Heliyon.

[53] Moyib O.K., Odunola O.A., Dixon A.G.O. (2007). SSR markers reveal genetic variation between improved cassava cultivars and landraces within a collection of Nigerian cassava germplasm. Afr. J. Biotechnol..

[54] Turyagyenda L.F., Kizito E.B., Ferguson M.E., Baguma Y., Harvey J.W., Gibson P., Wanjala B.W., Osiru D.S.O. (2012). Genetic diversity among farmer-preferred cassava landraces in uganda. Afr. Crop Sci. J..

[55] Pedri E.C.M., Hoogerheide E.S.S., Tiago A.V., Cardoso E.S., Pinto J.M.A., Santos L.L., Yamashita O.M., Rossi A.A.B. (2019). Genetic diversity of cassava landraces cultivated in northern Mato Grosso State, Brazil, using microsatellite markers. Genet. Mol. Res..

[56] Kamvar Z.N., Tabima J.F., Grünwald N.J. (2014). Poppr: An R package for genetic analysis of populations with clonal, partially clonal, and/or sexual reproduction. PeerJ.

[57] Rabbi I.Y., Kulakow P.A., Manu-aduening J.A., Dankyi A.A., Asibuo J.Y., Parkes E.Y., Abdoulaye T., Girma G., Gedil M.A., Ramu P. (2015). Tracking crop varieties using genotyping-by-sequencing markers: A case study using cassava (*Manihot esculenta* Crantz). BMC Genet..

[58] Yang X., Xu Y., Shah T. (2011). Comparison of SSRs and SNPs in assessment of genetic relatedness in maize. Genetica.

[59] Emanuelli F., Lorenzi S., Grzeskowiak L., Catalano V., Stefanini M., Troggio M., Myles S., Martinez-zapater J.M., Zyprian E., Moreira F.M. (2013). Genetic diversity and population structure assessed by SSR and SNP markers in a large germplasm collection of grape. BMC Plant Biol..

