# Supplementary figures and images for: Estimation of Genetic Diversity and Number of Unique Genotypes of Cassava Germplasm from Burkina Faso Using Microsatellite Markers
Source: Genes (Basel). 2024 Jan 5;15(1):73. doi: 10.3390/genes15010073 (PMC10815475; doi:10.3390/genes15010073)

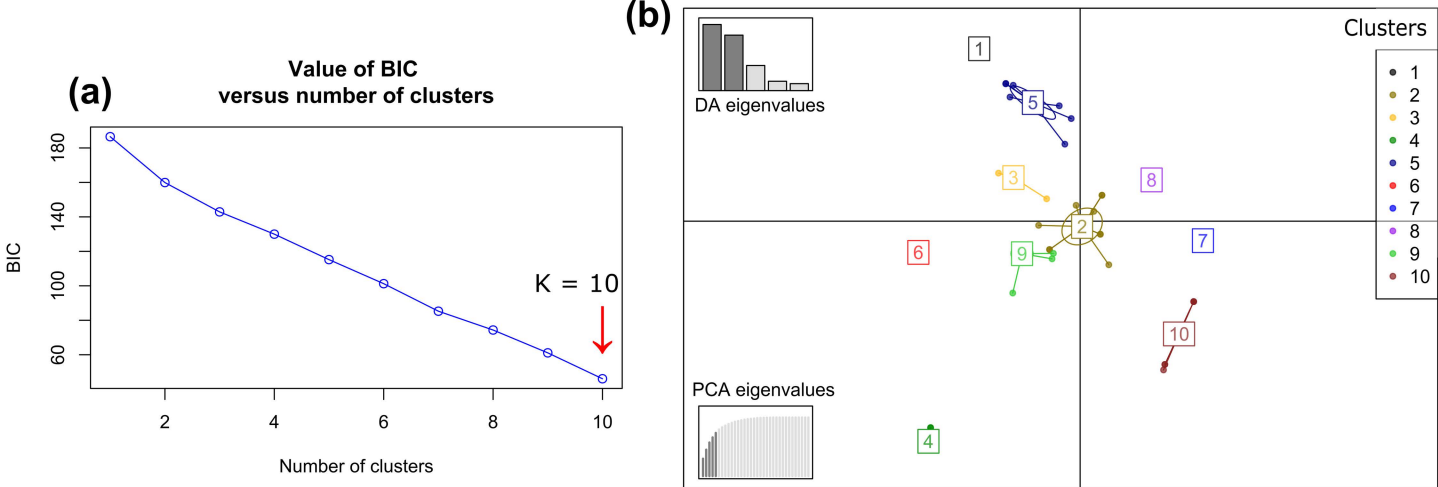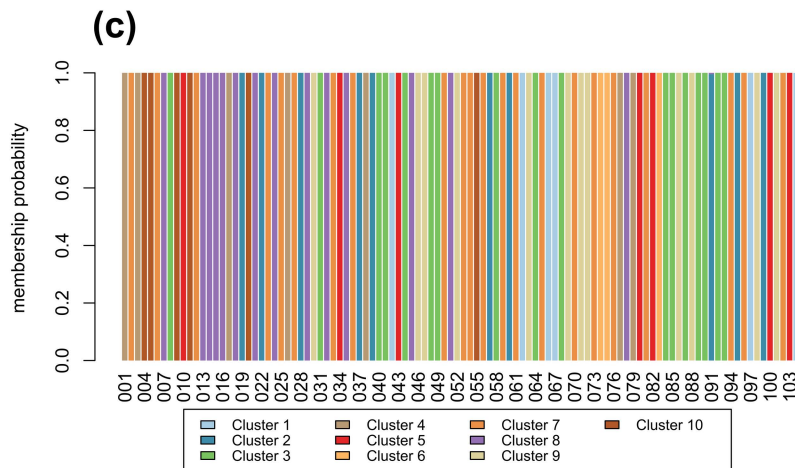

Supplement: Supplementary file 1 [file genes-15-00073-s001.zip › Figure S1.pdf]

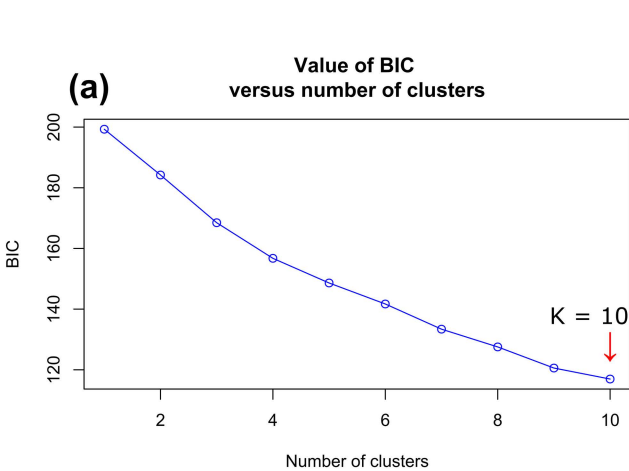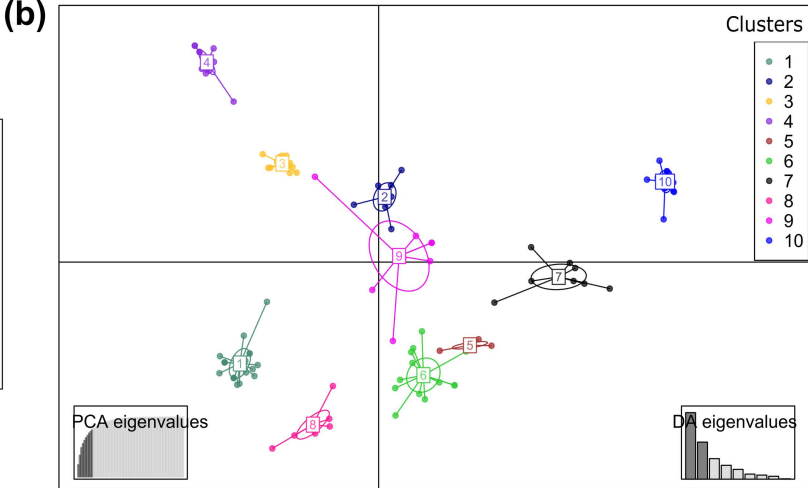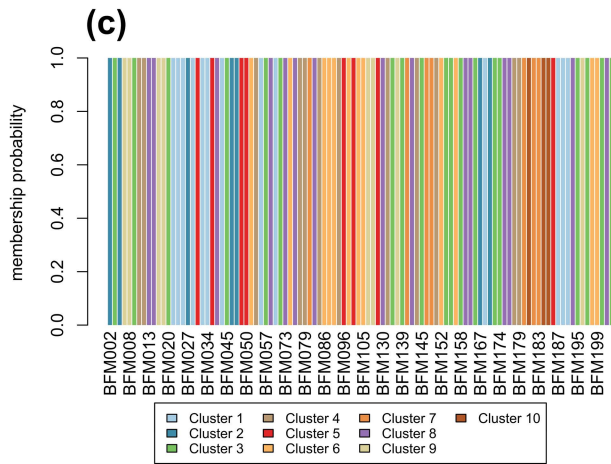

Supplement: Supplementary file 1 [file genes-15-00073-s001.zip › Figure S2.pdf]
